# Molecular detection of hypervirulent *Klebsiella pneumoniae* (hvKp) in Egyptian poultry

**DOI:** 10.1186/s12917-026-05339-5

**Published:** 2026-03-02

**Authors:** Zeinab S. Ahmed, Fatma Abdel-Kader, Dalia Hamza, Radwa Ashour

**Affiliations:** Department of Zoonoses, Faculty of Veterinary Medicine, Cairo University, P.O. Box 12211, Giza, Egypt

**Keywords:** Hypervirulent *Klebsiella pneumoniae* (hvKp), Virulence gene, Poultry, Egypt

## Abstract

The emergence of hypervirulent *Klebsiella pneumoniae* (hvKp) in poultry represents a significant and growing threat to both public health and the poultry industry. However, epidemiological data on hvKp in Egyptian poultry remain limited. In this One Health–focused study, 150 cloacal swabs from broiler chickens, 30 hand swabs from workers in direct contact with the birds, and 20 drinking water samples were collected from six poultry farms in Giza Governorate, Egypt. All samples were processed using standard microbiological methods, and the presence of *K. pneumoniae* was confirmed through molecular detection of the *gyrA* gene and the 16–23 S ITS region. Hypervirulent *K. pneumoniae* strains were characterized by multiplex PCR targeting key virulence determinants (*rmpA*,* rmpA2*,* iucA*,* iroB*, and *peg-344*) and capsular serotypes (K1 and K2). Additional virulence-associated markers (*entB*,* kfu*, and *mrkD*) were also evaluated to assess their pathogenic potential. *K. pneumoniae* was identified in 13.3% (20/150) of chicken samples, while exhibiting a prevalence of 10% in both workers (3/30) and water (2/20) samples. Of the 25 positive isolates, the most frequently identified hypervirulence genes were *iroB* (64%, *n* = 16) and *peg-344* (32%, *n* = 8), followed by *rmpA* (8%, *n* = 2) and *iucA* (8%, *n* = 2). The genes *mrkD* (100%, *n* = 25) and *entB* (92%, *n* = 23) were the most prevalent among all virulence genes. All positive samples carried at least one virulence gene. Notably, none of the samples tested positive for the K1, K2, or *rmpA2* genes. The detection of isolates harboring virulence-associated genes confirms the circulation of highly virulent *K. pneumoniae* strains within poultry environments, presenting substantial risks to animal health and indicating potential occupational exposure among farm workers. Strengthening biosecurity, hygiene measures, and routine surveillance programs in poultry farms is urgently needed to curb hvKp transmission and mitigate its potential public health impact.

**Supplementary Information:**

The online version contains supplementary material available at 10.1186/s12917-026-05339-5.

## Introduction

The genus *Klebsiella* comprises many species, but *Klebsiella pneumoniae* (Kp) is the most clinically significant for humans and animals. This bacterium is additionally frequently detected in the environment, including surface water, sewage, plants, and soil [1]. Kp can additionally be classified as a foodborne pathogen, as it has been detected in various food products, including shellfish, frozen foods, and fresh chickens [2]. Kp is known to cause severe nosocomial infections such as septicemia, pneumonia, and urinary tract infections [3]. Since Kp is ubiquitous, adopting a One Health approach is crucial for addressing its impact on humans, animals, and the environment [4]. Cases of hypervirulent *Klebsiella pneumoniae* (hvKp), a highly pathogenic form of Kp, have increased significantly in recent years, posing a significant public health risk to both humans and animals [5]. Moreover, hvKp is associated with high mortality rates, ranging from 3% to 42% [6]. The hvKp was first identified in Taiwan during the 1980s in a clinical case of tissue-invasive Kp infection in an immunocompetent individual. Since then, it has disseminated globally from Taiwan to many countries across America, Europe, Australia, and the Middle East [7, 8]. In both immunocompromised and healthy individuals, hvKp isolates are associated with a higher incidence of severe illnesses, including pyogenic liver abscesses, meningitis, urinary tract infections, necrotizing ascites, and endophthalmitis [9]. The pathogenicity of hvKp is linked to multiple virulence factors that enable evasion of the host’s innate immune defenses [10]. These factors include capsular antigen, the colibactin toxin, exopolysaccharides contributing to mucoviscosity, lipopolysaccharides (LPSs), adhesins, and iron uptake systems [10]. The production of biofilm is notably higher in hvKp strains compared to classical *K. pneumoniae* (cKp) strains, suggesting a role for biofilm formation in hvKp pathogenesis and its contribution to bacterial survival and emergence [11, 12]. Historically, hvKp isolates were identified through a positive string test; however, this method has proven less reliable in regions with low prevalence of hvKp infection [5]. Recently, reliable hypervirulence biomarker genes, including *peg-344*, *iroB*, *iucA*, *rmpA*, and *rmpA2*, have been identified, providing essential insights into the bacterium’s pathogenesis [13, 14]. The *peg-344* gene encodes a putative transporter protein that may assist in nutrient acquisition or toxin secretion [15]. The salmochelin siderophore biosynthesis (*iroB*) gene functions in iron capture, whereas the aerobactin siderophore biosynthesis (*iucA)* gene facilitates its delivery to the bacterium [16]. Additionally, the regulator of mucoid phenotype (*rmpA*) and regulator of mucoid phenotype A2 (*rmpA2*) genes regulate the expression of capsule polysaccharides, which are crucial for resistance to phagocytosis and mucoviscosity [17]. While most research on hvKp has focused on human patients, limited attention has been given to its occurrence in healthy animals, particularly in Egyptian poultry, highlighting a significant gap in regional public health and zoonotic pathogen surveillance [18]. In poultry, Kp is a primary causative agent of pulmonary infections, resulting in substantial economic losses in Egypt, as it accounts for approximately 30% of annual mortality in broilers and chicks [19–21]. Although previous studies have confirmed the presence of Kp in poultry within Egypt (Ismailia, Alex, and Kafr Elsheikh governorates) [20, 22, 23], there remains a critical lack of research on its epidemiology and the zoonotic risks associated with hvKp. Consequently, this study aimed to investigate the prevalence of hvKp in chickens raised for human consumption at the farm level across Giza governorate, Egypt. It further seeks to analyze key pathogenicity-related genes as mannose-resistant *Klebsiella*-like hemagglutinin D (*mrkD)*, enterobactin B *(entB)*,* Klebsiella* ferric uptake (*Kfu*), and capsular serotypes (K1/K2) to provide deeper insights into the role of Kp in infections, thereby enhancing the understanding of its virulence and epidemiological significance.

## Materials and methods

### Collection of samples

A six commercial broiler chicken farms were selected in the Giza governorate, Egypt. These farms were chosen using convenience sampling based on accessibility, and all were confirmed to be raising chickens for human consumption. From these farms, A 150 cloacal swabs were collected from individual broiler chickens, which were selected randomly from the poultry houses. Sampling was limited to healthy-appearing birds to ensure the findings represented the *K. pneumoniae* found in the normal commercial poultry population, avoiding samples from clinically sick animals. Also 30 hand swabs were collected from workers who had contact with the birds and 20 drinking water samples from the chicken drinking sources. All samples were analyzed for the presence of Kp. Cloacal and hand swabs were taken using sterile swabs and placed in sterile saline (0.9% NaCl) (Oxoid, England). Drinking water was collected in sterile 1-liter glass bottles. Samples were transported in iceboxes to the Laboratory of the Zoonoses Department, Faculty of Veterinary Medicine, Cairo University, for further analysis.

### Isolation and identification of *Klebsiella* spp

#### Microbiological identification

Cloacal and hand swab samples were inoculated into brain heart infusion (BHI) broth (Oxoid, UK) and incubated at 37 °C for 24 h to promote the growth of *Klebsiella*. The inoculated samples were then plated onto MacConkey agar (Oxoid, Hampshire, UK) and incubated at the same temperature. For water samples, the membrane filtration method was applied, in which 100 mL of each sample was passed through a sterile membrane filter with a pore size of 0.45 μm. The filters were carefully placed on MacConkey agar plates and incubated at 37 °C for 18–24 h. Presumptive *Klebsiella* spp. colonies, identified as pink to purple, mucoid, and measuring 2–3 mm in diameter, were purified by subculture on MacConkey agar to obtain pure cultures [24]. To ensure accurate identification beyond colony morphology, the pure cultures were subjected to a comprehensive series of conventional biochemical tests, including catalase, oxidative-fermentative, indole, methyl red, Voges-Proskauer, citrate utilization, urea hydrolysis, carbohydrate fermentation, and triple sugar iron (TSI) agar tests [22], with all reagents obtained from Oxoid (Hampshire, UK). Following identification, isolates were grown in BHI broth for further examination.

#### Molecular analysis

##### DNA extraction

DNA extraction was performed using the boiling method as described in [23]. A 1.5 mL culture of a single colony in BHI broth was incubated overnight at 37 °C and then centrifuged at 1200 rpm for 10 min to form a pellet. The pellet was resuspended in 150 µL of sterile distilled water and heated at 100 °C for 15 min using a heat thermo-blocker to lyse the cells. It was then stored at -20 °C overnight before undergoing a second centrifugation at 12,000 rpm for 10 min. The supernatant containing the extracted DNA was used as a template for PCR analysis and stored at -20 °C for future use.

##### Molecular identification of *Klebsiella spp.* isolates

The *gyrA* gene, a highly conserved genetic marker, was used for the molecular identification of *Klebsiella* genus isolates by uniplex polymerase chain reaction (PCR) [25]. Additionally, PCR was employed to distinguish species within the confirmed *Klebsiella* isolates, targeting the 16–23 S ITS gene for Kp [26] and the *pehX* gene for *K. oxytoca* [27]. Table 1 presents the primer sequences, amplicon sizes, and annealing Temp. for these genes.

**Table 1:** The sequence of species-specific primers and their annealing temperatures

Target	Gene	Primer sequence (5’ 3’)	Ann. Temp.	bp	References
*Klebsiella* genus	*gyrA*	F: CGC GTA CTA TACGCC ATG AACGTA R: ACC GTT GAT CACTTC GGTCAGG	55 °C	441	[25]
*Klebsiella* spp	16–23 S ITS (Kp)	F: ATTTGAAGAGGTTGCAAACGAT R: TTCACTCTGAAGTTTTCTTGTGTTC TCTTGTGTTC	55 °C	130	[26]
	*pehX (K. oxytoca*)	F: GAT ACG GAG TATGCC TTT ACGGTG R: TAG CCT TTA TCAAGC GGA TACTGG		343	[27]

### Molecular detection of virulence and hypervirulence genes

A multiplex PCR assay was used to determine the capsular type of the isolates specifically targeting genes associated with capsular serotypes K1 and K2 as well as the presence of additional virulence-associated markers (*entB*,* Kfu*,* and mrkD*) [28, 29]. Furthermore, five uniplex PCR assays were performed to detect the hypervirulence biomarker genes: *iucA*,* iroB*,* peg-344*,* rmpA*, and *rmpA2* [30]. Isolates were classified as hypervirulent (hvKp) if they carried at least one of these biomarker genes, following the criteria described by Russo et al. 2018 [31].

PCR was conducted in a total volume of 25 µL, consisting of 5 µL of DNA template, 5 µL of 5×TaqMaster mix (Jena Bioscience, Germany), and 1 µL of each forward and reverse primer (10 pmol/µL), with the final volume adjusted to 25 µL using PCR-grade water (Jena Bioscience, Germany). Each set of reactions included positive control, while the negative control consisted of nuclease-free water used in place of the DNA sample in the PCR mixture. The amplified products were separated by electrophoresis on 1.5% agarose stained with 0.5 µg/mL ethidium bromide and visualized under a UV illumination system. Gel images were captured using the GelDoc 1000 fluorescent imaging system (Bio-Rad) and analyzed with Gel-Pro Analyzer^®^ version 4 (Media Cybernetics, Silver Spring, MD, USA). Details of the primer sets, amplicon sizes and annealing Temp. for each gene are summarized in Table 2.

**Table 2:** The sequence of virulence/hypervirulence gene primers and their annealing temperatures

Target	Gene	Sequence (5’ 3’)	Ann. Temp.	bp	References
Capsular Serotype	K1	F: GGT GCT CTT TAC ATC ATTGC R: GCA ATG GCC ATT TGC GTTAG	60 °C	1283	[28, 29]
	K2	F: CAA CCA TGGTGG TCG ATTAG R: TGG TAG CCATAT CCC TTTGG		531	
Virulence genes	*mrkD*	F: AAG CTA TCG CTG TAC TTCCGGCA R: GGC GTT GGCGCT CAG ATAGG		340	
	*entB*	F: GTC AAC TGGGCC TTT GAGCCGTC R: TAT GGG CGTAAA CGC CGGTGAT		400	
	*Kfu*	F: GGC CTT TGT CCA GAG CTACG R: GGG TCT GGC GCA GAG TATGC		638	
Hypervirulent genes	*iucA*	F: AATCAATGGCTATTCCCGCTG R: CGCTTCACTTCTTTCACTGACAGG	59 °C	239	[30, 31]
	*iroB*	F: CAAAAAAGCAGCAGCAGAGGC R: TCACTGGCGGAATCCAACAC		585	
	*rmpA*	F: GTAGTTAATAAATCAATAGCAAT R: CAGTAGGCATTGCAGCA	50 °C	332	
	*rmpA2*	F: GTGCAATAAGGATGTTACATTA R: GGATGCCCTCCTCCTG		430	
	*peg-344*	F: AAAGGACAGAAAGCCAGTG R: CCAATGACGAGGGGGATAATC	53 °C	411	

### Phenotypic confirmation of HvKp

All hypervirulent isolates identified by molecular methods were confirmed phenotypically using the string test, following the procedure described by Fang et al. (2004) [32]. A positive result was defined as the formation of a viscous string greater than 5 mm in length.

### Statistical analysis

The data were analyzed using SPSS version 18.0, with a *p*-value < 0.05 considered statistically significant. Chi-square (χ²) testing was applied to compare multiple groups of categorical samples.

## Results

### Occurrence of Kp in chickens, workers, and drinking water

All phenotypically recovered isolates were confirmed as *Klebsiella* spp. based on the presence of the highly conserved *gyrA* gene, while species-specific identification of *K. pneumoniae* was verified using the 16–23 S ITS region. Of the 200 samples analyzed, 25 were positive for both markers, whereas all were negative for the *pehX* gene. Among the 150 chicken samples, 20 (13.3%) were confirmed positive for *K. pneumoniae*. Similarly, 3 of 30 hand swabs from workers (10%) and 2 of 20 drinking water samples (10%) tested positive (Table 3). A chi-square test of independence revealed no significant association between sample source and *K. pneumoniae* detection rate (χ² = 0.38, *p* = 0.826).

**Table 3:** Occurrence of Kp and HvKp in chickens, contact workers, and drinking water

Samples	No. examined	No. of Kp (%)	95% CI	No. of hvKp (%)	95% CI
Chicken	150	20 (13.3%)	8.7–19.7	16 (10.7%)	06.58–16.72
Contact workers	30	3 (10%)	2.66–26.42	2 (6.7%)	0.80- 22.37
Drinking Water	20	2 (10%)	1.57–31.32	0	00.0- 18.98
Total	200	25 (12.5%)	8.56–17.85	18 (9.0%)	5.7- 13.85
*χ*^*2*^ value	**-**	0.381	-	2.686	-
*p* value	**-**	0.826	-	0.261	-

*χ*^2^: Chi-Square. Significant was set at *p* < 0.05

*CI* confidence interval

### Distribution of virulence and hypervirulent genes among different sample types of Kp

Table 4 provides a comprehensive overview of the distribution of virulence and hypervirulent genes among different Kp sample types detected through uniplex and multiplex PCR assays. Among the 20 chicken samples, all were positive for *mrkD* (100%), and 18 (90%) were positive for *entB*. Additionally, four samples (20%) carried the *Kfu* gene, two (10%) harbored the *iucA* gene, 14 (70%) contained the *iroB* gene, six (30%) were positive for *peg-344*, and two (10%) carried the *rmpA* gene. None of the chicken samples contained the K2, K1, or *rmpA2* genes. Out of 20 chicken samples, 16 (80%) were classified as hypervirulent (hvKp), being positive for at least one of the five virulence marker genes (*iucA*,* iroB*,* peg-344*,* rmpA*,* and rmpA2*) [31]. Among these, isolates exhibiting a positive string test were noted, providing additional support for their hypervirulent phenotype [32].

**Table 4:** The distribution of virulence and hypervirulent genes among different sample types

Virulence genes								Hypervirulent genes				
		Capsular serotype			Iron uptake gene	Biofilm gene	Siderophores			Hypermucoviscosity-related gene		Virulence-enhancing gene
Samples	Total No	K1	K2		*Kfu*	*mrkD*	*entB*	*iucA*	*iroB*	*rmpA*	*rmpA2*	*peg-344*
Chicken	20	0 (0%)	0 (0%)		4 (20%)	20 (100%)	18 (90%)	2 (10%)	14 (70%)	2 (10%)	0 (0%)	6 (30%)
Contact workers	3	0 (0%)	0 (0%)		0 (0%)	3 (100%)	3 (100%)	0 (0%)	2 (66.6%)	0 (0%)	0 (0%)	2 (66.6%)
Drinking Water	2	0 (0%)	0 (0%)		0 (0%)	2 (100%)	2 (100%)	0 (0%)	0 (0%)	0 (0%)	0 (0%)	0 (0%)
X2	-	-			-	-	-	-	0.013	-	-	1.546
Total	25	0 (0%)	0 (0%)		4 (16%)	25 (100%)	23 (92%)	2 (8%)	16 (64%)	2 (8%)	0 (0%)	8 (32%)
*P*-value (chi-square)				< 0.001								

X2: Chi-square test. A *p*-value < 0.05 is considered statistically significant

All three worker samples were positive for both *mrkD* (100%) and *entB* (100%), while two samples (66.6%) carried the *iroB* and *peg-344* genes. None of the workers’ samples contained the K1, K2, *Kfu*, *iucA*, or *rmpA2* genes. Two of the three worker hand swabs were identified as carrying hypervirulent genes and positive string tests. Both water samples were positive for *mrkD* (100%) and *entB* (100%); however, neither contained any of the other genes under investigation.

### Gene profile of biomarkers among virulence and hypervirulent Kp isolates

**Fig. 1:**
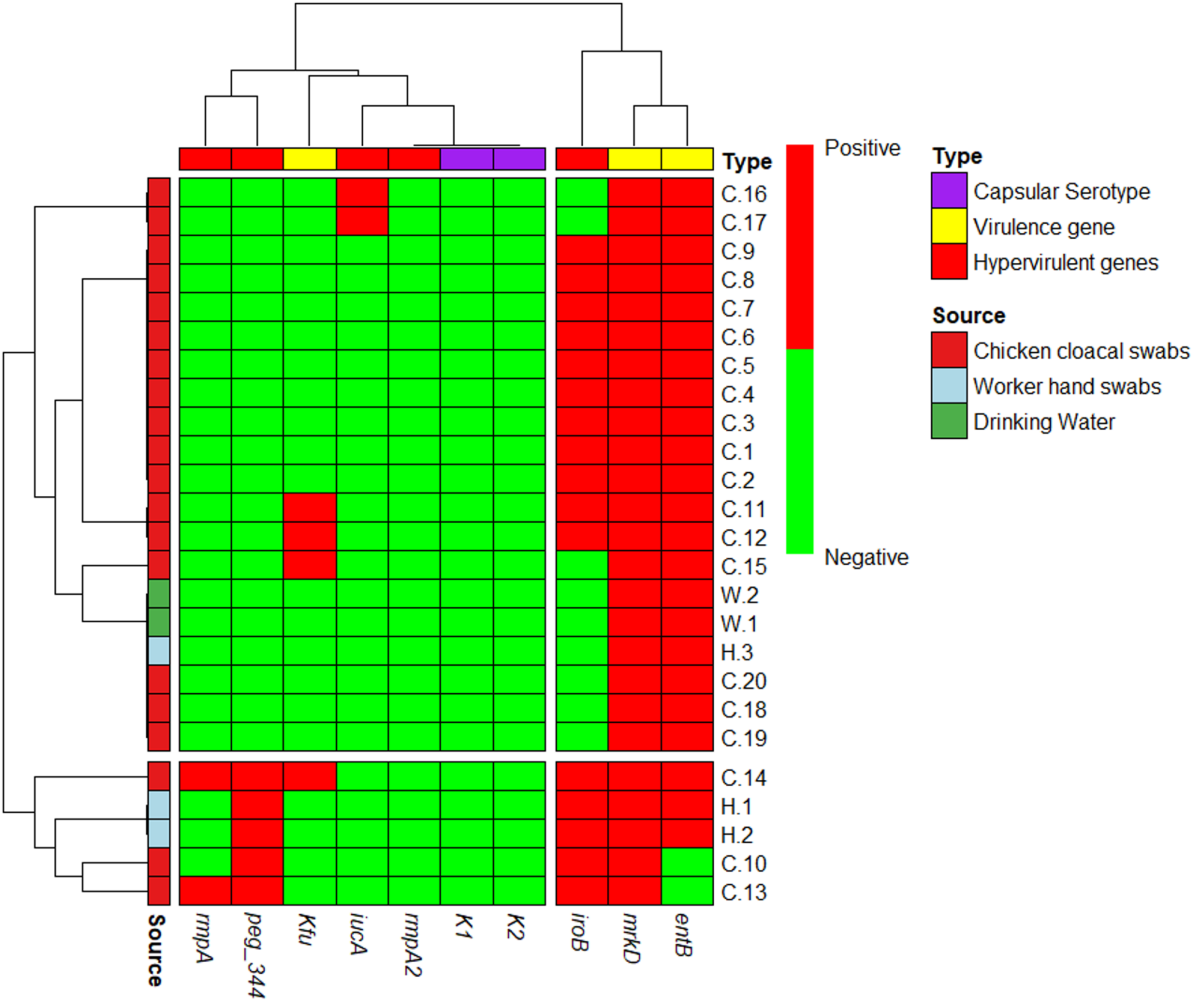
Hierarchically clustered heatmap showing the distribution of hypervirulent genes and virulence genes of 25 KP strains isolated from Chickens (*n* = 20), worker hand swabs (*n* = 3), and Drinking water (*n* = 2). The map plotted the hyper-virulence genes (*iucA*,* iroB*,* Peg344*,* rmpA*,* rmpA2*), classical-virulence genes (*mrkD*,* entB*,* kfu*), and Capsular serotype (K1, K2) as positive (red) and negative (green)

Table 5 and Fig. 1 illustrate the genetic profiling of *K. pneumoniae* isolates across chicken, worker, and water samples. Hierarchical clustering (Fig. 1) revealed that the adhesin gene *mrkD* and the siderophore gene *entB* were ubiquitously present across almost all lineages. In chicken samples, genetic diversity was high. Six isolates carried a profile of *mrkD*, *entB*, and *iroB*, while others displayed more complex hypervirulent profiles. Notably, distinct clusters emerged containing *peg-344* and *rmpA*: one sample harbored *mrkD*, *iroB*, *peg-344*, and *rmpA*, while another included *entB* and *Kfu* in addition to this set. Conversely, water samples (W.1, W.2) formed a distinct low-virulence cluster, testing positive only for *mrkD* and *entB*. Among worker samples, a potential transmission link was observed; two isolates (H.1, H.2) clustered closely with high-virulence chicken isolates (e.g., C.13, C.14), sharing the *mrkD*,* entB*,* iroB*, and *peg-344* profile. Significantly, despite the presence of hypervirulence-associated genes, all isolates tested negative for capsular serotypes K1 and K2.

**Table 5:** Gene profile of virulence and hypervirulent genes in different samples

samples type	Gene profile	No. of isolates
Chickens	*mrkD*,* entB*,* iroB*,* peg-344*	3
	*mrkD*,* entB*,* iroB*	6
	*mrkD*,* iroB*,* peg-344*	1
	*mrkD*,* entB*,* Kfu*,* iroB*	2
	*mrkD*,* iroB*,* peg-344*,* rmpA*	1
	*mrkD*,* entB*,* Kfu*,* iroB*,* peg-344*,* rmpA*	1
	*mrkD*,* entB*,* Kfu*	1
	*mrkD*,* entB*,* iucA*	2
	*mrkD*,* entB*	3
Contact workers	*mrkD*,* entB*,* iroB*,* peg-344*	2
	*mrkD*,* entB*	1
Drinking water	*mrkD*,* entB*	2

## Discussion

*Klebsiella pneumoniae* is an opportunistic pathogen capable of infecting the respiratory system of chickens, leading to substantial production losses and increased mortality in the poultry industry [33]. In the present study, *K. pneumoniae* was isolated from multiple sources, with the highest prevalence recorded in poultry (13.3%). This finding is consistent with a previous report from Egypt, which documented a similar detection rate [34], yet it remains considerably lower than the 67.2% prevalence reported in Ethiopia [33]. The detection of *K. pneumoniae* in both drinking water and worker hand swabs further underscores an important public health concern. Contaminated water, often resulting from agricultural runoff, sewage, and animal waste, can serve as a potent vehicle for spreading the bacterium to humans and animals, particularly in areas with poor sanitation [35]. The virulence profiles of the recovered isolates were also examined, with attention to hypervirulent strains. Poultry samples exhibited the highest prevalence of virulence determinants, followed by human samples, while water isolates showed the lowest.

Capsular serotyping is often used to identify lineages associated with hypervirulent *K. pneumoniae* (hvKp). Among more than 78 recognized capsular types, K1 and K2 are most strongly linked to hvKp and are known to enhance resistance to phagocytosis [36]. In this study, none of the *K. pneumoniae* isolates were identified as K1or K2 serotypes. This contrasts with Osama et al. (2023) [37], who reported a high prevalence of K2 among hvKp isolates, but aligns with other studies documenting absence of K1 serotype [37, 38]. Importantly, the presence of K1 or K2 alone does not account fully for hypervirulence, as these serotypes often coexist with additional virulence factors [36, 39]. The relatively low prevalence of *rmpA* (8%) and complete absence of *rmpA2* contrasts with reports from other reports [28, 40] where these genes are strongly associated with hypermucoviscosity [41]. Notably, the absence of classical hypervirulent capsular serotypes (K1, K2) and *rmpA2* indicates that hypervirulence in these isolates may be multifactorial trait influenced by novel and unidentified virulence factors and achieved through alternative mechanisms, such as the siderophore systems *iroB* and *entB* rather than capsule-mediated mucoviscosity alone [37].

Iron acquisition systems (siderophores) are essential for *K. pneumoniae* survival and pathogenicity [42]. Among the three major siderophore systems examined in this study (enterobactin, aerobactin, salmochelin), the *entB* gene (enterobactin) exhibited the highest prevalence (92%), in agreement with previous reports identifying *entB* as the most widespread siderophore gene among *K. pneumoniae* isolates [37, 43, 44]. Also, the *mrkD* gene was detected in all isolates, confirming its universal presence and supporting earlier findings that highlight its role in biofilm formation and persistence on both host tissues and environmental surfaces [41, 45]. Furthermore, the *Kfu* gene, which contributes to iron uptake, was identified in 20% of chicken isolates but was absent from worker and water samples. This prevalence is lower than that reported in other studies [46, 47]. The molecular analysis, visualized in the heatmap, provides compelling evidence of a strong link between the chicken and worker samples. Interestingly, the isolates obtained from workers harbored a virulence-associated gene profile (*rmpA*,* peg-344*,* iroB*,* mrkD*,* entB*) that is strikingly similar to chicken isolates. This close similarity may indicate a shared genetic background and suggests a possible epidemiological connection between poultry and occupationally exposed individuals. Such findings lend support to the hypothesis that repeated or prolonged contact with poultry could contribute to zoonotic transmission. Nevertheless, the cross-sectional nature of the current study limits definitive conclusions regarding transmission pathways. Further investigations incorporating whole-genome sequencing, phylogenetic analysis, and longitudinal sampling of both animal and human populations are essential to clarify the directionality, frequency, and public health implications of this potential interspecies transmission.

Human exposure may occur through direct contact, handling contaminated materials, or cross-contamination. These findings emphasize the urgent need for a comprehensive One Health approach to mitigate the dissemination of Kp within Egypt’s poultry sector. Strengthening farm biosecurity, improving water sanitation, and enhancing public health surveillance are crucial. Future studies should integrate data on both virulence and antimicrobial resistance to improve management. Additionally, larger, farm-matched studies employing advanced statistical methods are recommended to accurately map the spatial distribution and source-to-host transmission of hvKp in the poultry environment.

## Conclusion

This study establishes a foundational framework for molecular surveillance of *Klebsiella pneumoniae* in Egyptian poultry farms, revealing the circulation of virulence-associated genes among isolates recovered from chickens, farm workers, and drinking water. While multiple markers linked to hypervirulence were identified, the absence of classical hypervirulent capsular types, together with the lack of whole-genome data, limits definitive interpretation of strain lineage, transmission dynamics, and clinical significance. Consequently, comprehensive future investigations integrating whole-genome sequencing, plasmid characterization, and broader, longitudinal sampling are required to clarify strain-level relatedness and to more accurately evaluate the zoonotic risk posed by poultry-associated *K. pneumoniae*. Until such data is available, the reinforcement of biosecurity measures, improved hygiene practices, and sustained surveillance represent essential strategies to mitigate the dissemination of opportunistic pathogens within poultry production systems.

## Supplementary Information

Supplementary Material 1.

## Data Availability

All data generated or analyzed in this study are included in this published article.

## Abbreviations

BHI: Brain Heart Infusion
entB: Enterobactin B
Hv: Hypervirulent
K2: Capsular serotype gene K2
Kfu: Klebsiella ferric uptake
MagA: Mucoviscosity-Associated Gene A
mrkD: Mannose-resistant Klebsiella-like hemoagglutinin D
PCR: Polymerase Chain Reaction
rmPA: Regulatory Mucoid Phenotype A
iucA: Aerobactin siderophore biosynthesis
iroB: Salmochelin siderophore biosynthesis
peg-344: Putative Transporter
rmPA: Regulator of mucoid phenotype A
rmPA2: Regulator of mucoid phenotype A2
VETCU-IACUC: Veterinary Medicine, Cairo University-Institutional Animal Care and Use Committee
