# Supplementary material for: Molecular detection of hypervirulent Klebsiella pneumoniae (hvKp) in Egyptian poultry
Source: BMC Vet Res. 2026 Mar 2;22:208. doi: 10.1186/s12917-026-05339-5 (PMC13059463; doi:10.1186/s12917-026-05339-5)
Supplement: Supplementary file 1 — Supplementary Material 1. [file 12917_2026_5339_MOESM1_ESM.pdf]

The Institutional Animal Care and Use Committee

Vet. CU. IACUC

Letter of IACUC Protocol Approval

Vet CU110520251142

From One Health view: Molecular Detection of Virulence genes of Hypervirulent Klebsiella pneumoniae in Egyptian poultry .

Department of Zoonoses Depart ment Faculty of veterinary medicine Cairo University .

Dear **Prof.Dr. Dalia A. Hamza** (Fatma Abdel-Kader ;Zeinab Ahmed ;Radwa Ashour)

The Institutional Animal Care and Use Committee (IACUC) has **APPROVED** the above referenced Animal Use Protocol (AUP).

Date of Approval : 11/05/2025

Date of Expiration : 10/05/2026

During this one year approval period, annual reviews are required. The IACUC staff will make every effort to send the Principal Investigator annual reminders. However, the Principal Investigator is responsible for submitting an Annual Review in advance of the annual review due dates to ensure continuing IACUC approval. It is very important that these deadlines are not missed. Failure to submit an Annual Review on time will result in in the termination of the protocol.

To continue this research beyond the three years approval period, a new protocol submission will be required. To avoid a lapse in IACUC approval, it is essential that the completed renewal protocol be submitted and approved by the IACUC prior to its expiration date.

Any activities conducted under the protocol after expiration will be in direct violation of IACUC policies. It is the responsibility of the Principal Investigator to notify the IACUC of any proposed changes regarding the work described within this protocol. The Principal Investigator agrees that no such changes will be implemented until approved by the IACUC, except where necessary to eliminate apparent immediate hazards to person(s) and/or animal(s).

Prof. Fathy F. Mohamed

Vet.CU.IACUC Chair

iacuc@vet.cu.edu.eg
